# Deltamethrin toxicological profile of peridomestic *Triatoma sordida* in the North of Minas Gerais, Brazil

**DOI:** 10.1186/s13071-015-0873-7

**Published:** 2015-05-08

**Authors:** Grasielle Caldas DÁvila Pessoa, Marcos Takashi Obara, João G Rezende, Bernardino Vaz de Mello, Marcela Lencine Ferraz, Liléia Diotaiuti

**Affiliations:** Laboratório de Triatomíneos e Epidemiologia da Doença de Chagas/CPqRR/FIOCRUZ, Centro de Pesquisas René Rachou, Fundação Oswaldo Cruz, Av. Augusto de Lima 1715, Barro Preto, 29190-002 Belo Horizonte, MG Brazil; Laboratório de Entomologia, Universidade Federal de Brasília, Brasília, DF Brazil; Gerência Regional de Saúde de Montes Claros, Montes Claros, MG Brazil; Secretaria de Saúde do Estado de Minas Gerais, Belo Horizonte, MG Brazil

**Keywords:** Triatominae, *Triatoma sordida*, Minas Gerais, Insecticide resistance, Deltamethrin

## Abstract

**Background:**

In general, there was a large reduction in the occurrence of cases of Chagas disease in the last decades in Brazil. However, despite all of these efforts, there have been various reports of persistent reinfestations of *T. sordida* in a large part of the state of Minas Gerais, for reasons still little investigated. Thus**,** this purpose of this study was to characterize the deltamethrin susceptibility profile of peridomestic *T. sordida* populations from North of Minas Gerais – Brazil.

**Methods:**

Susceptibility to deltamethrin was assessed in seventeen peridomestic populations of *T. sordida* from North region of Minas Gerais, Brazil. Serial dilutions of deltamethrin in acetone (0.2 μL) were topically applied in first instar nymphs (F1, five days old, fasting, weight 1.2 ± 0.2 mg). Dose response results were analyzed with POLO program, determining the lethal doses, slope and resistance ratios (RR).

**Results:**

Susceptibility profile characterization of *T. sordida* populations revealed resistance ratios (RR_50_) ranging from 2.50 to 7.08.

**Conclusions:**

In fact, we know very little about the real impact of the resistance ratios obtained in the laboratory bioassays on the effectiveness of the vector control activities in the field. Thus, we prefer to refer to the populations with RR > 5 as populations with altered susceptibility. For these populations, the realization of laboratory and field trials, simultaneous and complementary, permitting the evaluation of both, is recommended.

## Background

Chagas disease (CD), or American trypanosomiasis, is a public health concern in most Latin American countries. About 7 million people are infected by causative agent of this disease, Trypanosoma cruzi (Kinetoplastida; Trypanosomatidae) [1]. In Brazil, it has been estimated that approximately 1.8 to 2.5 million people are currently infected [2]. Considering the absence of a vaccine and the limited efficacy of the currently available parasiticide drugs, the main approaches to control are based on insecticidal control of insect vectors.

With the priorisation of the areas of *Triatoma infestans*, the prevalence of CD was reduced from approximately 18 million to the current 9 million, in little more than 15 years [2]. A marked reduction in the distribution of domestic vector transmission of the disease, by T*. infestans*, was also achieved, resulting in the interruption of the vector transmission of the disease by *T. infestans* in Brazil [3]. The recolonization of the areas originally occupied by *Triatoma infestans*, by secondary species, became a reality, bringing out the *Triatoma sordida. T. sordida* is currently considered as a possible substitute to the present domestic vector *T. infestans* in the transmission of *T. cruzi*. The process of domiciliation for *T. sordida* may be associated with a previous eradication of *T.infestans*, considering the better ability to obtain blood meals in the latter species, associated at the rarity of mixed populations where these two species occur in simpatry [4]. In Central Brazil, *T. sordida* is a triatomine that represents the greatest risk for the transmission of Chagas Disease [5].

*T. sordida* is a tritomine endemic in the savanna areas, living mainly under the bark of trees that remain preserved in the process of forming fields and pastures. Some biological characteristics of this triatomine, such as its resistance capacity to fasting, its ease in adapting to different hosts and relative mobility (mainly of the adults) facilitates its process of dispersion and colonization of artificial ecotopes [6-12]. The frequency with which *T. sordida* has been found in peridomicile and intradomicile environments, has characterized it as a semidomestic species [13]. In spite of the spraying difficulties and the low permanence of the pyrethroid insecticide in peridomiciliar environments, the existence of just one annual triatomine cycle and the slowness of original population reconstruction suggest that one annual spraying is sufficient for the control of this triatomine [14].

In general, there was a large reduction in the occurrence of cases of CD in the last decades in Brazil. This was possibly due to epidemiological vigilance, linked to the chemical control activities of the Chagas Disease Control Program as well as the improvement of socioeconomic factors in the rural areas - income improvement, habitation improvements, electricity, access to health and education [15]. However, despite all of these efforts, there have been various reports of persistent reinfestations of *T. sordida* in a large part of the state of Minas Gerais, for reasons still little investigated.

In this context, studies investigating a possible insecticide resistance in Brazilian *T. sordida* has been made. The susceptibility of 11 populations of *T. sordida*, collected in the states of Goiás, (GO), Mato Grosso (MT), and Mato Grosso do Sul (MS) to deltamethrin, revealed high levels of susceptibility (RR_50_ 1.19 to 2.26) [16]. The evaluation of susceptibility to deltamethrin of 103 populations of *T. sordida* collected in the Triângulo, Central and Northern part of the state of Minas Gerais, showed just one resistant population (RR_50_ 6.5) [17]. Thus, the objective of this present work was to characterize the deltamethrin toxicological profile of peridomestic *T.sordida* in the Northern region of Minas Gerais, originating from the areas with persistent reinfestations.

## Methods

### Insect sampling

The study was carried out in the Northern region of Minas Gerais (Brazil), area which presents the largest index of poverty in the State. Similar in many aspects to the Brazilian Northeast, this region shows extensive areas of transition between the savanna and the caatinga. In the 1980s, the cotton culture expanded representing nearly all of the agricultural investment in the region. The only reason deforestation did not reach the entire area was because of the cotton culture failure resulting from the introduction of pests and lack of financial support. This stimulated the migration of the rural population from areas with a high prevalence of Chagas disease (CD), to urban centers. For the subsisting rural population remained the possibility of using residual forests for the production of charcoal, or land sale and of physical labor for the reforestation with eucalyptus (growing in recent years in the area) [18].

**Figure 1:**
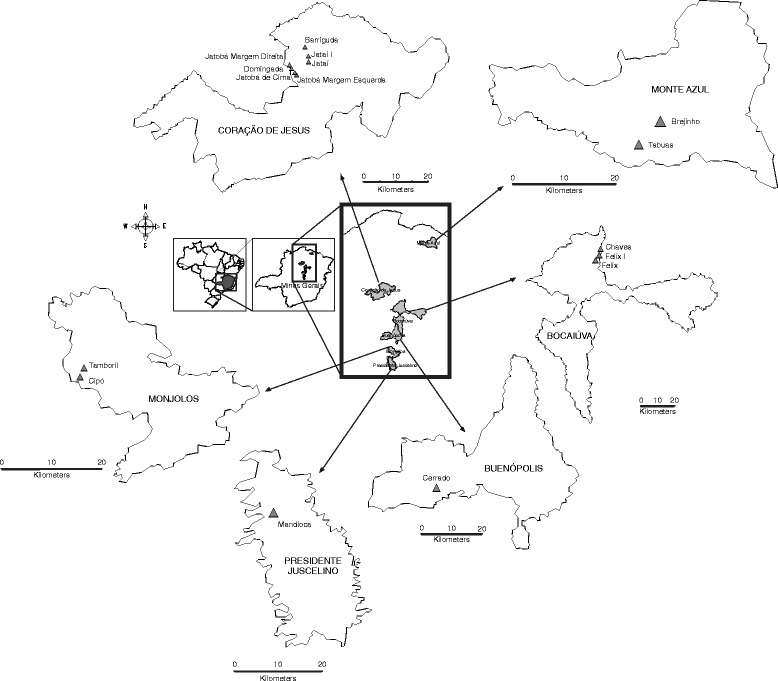
Map of Minas Gerais showing study sites where *T. sordida* were collected.

The study triatomines populations were manually collected, without using a dislodging agent, in the peridomiciles in endemic areas of the North of Minas Gerais (Monte Azul - 16° 13′ 01″ S 44° 54′ 21″ O, Monjolos - 20° 1′ 29″ S, 48° 56′ 27″ W, Buenópolis - 17° 52′ 22″ S, 44° 10′ 48″ W, Presidente Juscelino - 18° 38′ 13″ S 44° 03′ 28″ O, Coração de Jesus 16° 41′ 06″ S 44° 21′ 54″ O, Bocaiuva 17° 06′ 28″ S 43° 48′ 54″ O), in which the Chagas Disease Control Program were performed systematic applications of insecticides with residual action in the last 29 years (Figure 1).

### Chemicals

Technical grade Deltamethrin (99.1%) used for bioassay was obtained from Bayer CropScience, Brazil. The analytical grade acetone used for dilutions was purchased from Merck, Germany.

### Dose–response bioassays

The susceptibility reference lineage (SRL) of *T. sordida* came from Uberaba (19° 44′ 52″ S 47° 55′ 55″ O), preserved in the laboratory for more than 20 years, without contact with insecticide and inclusion of external material was used [19].

Serial dilutions of deltamethrin in acetone were prepared. For each concentration, three repetitions were carried out with ten first instar nymphs of F1 generation (five days old, fasting, weight of 1.2 ± 0.2 mg). The treatment consisted of the application of 0.2 μL of insecticide dilution on the dorsal abdomen, according to the World Health Organization [20] and Pessoa [21] procedures, with the aid of a Hamilton mycro-syringe mounted on a repeating dispenser. For each population, a minimum of eight doses of insecticide active ingredient (a.i.) ranging from 0.01 to 4.0 ng and killing between >0% to <100% of the individuals, were applied per insect. Acetone was applied to the control group. The mortality was assessed 72 hours after application and it was determined by the inability or lack of coordination of the nymphs to move from the center to the edge of the filter paper (7 cm diameter). Signs of paralysis and lack of response to external stimuli was considered as well. During and after the experiment, the insects were kept under controlled conditions of temperature and humidity (25°C ± 1°C; 60% ± 10% RH). Probit analysis [22] was performed on mortality data using POLO program [23] to determine the LD_50_. Dosages were expressed as nanograms of active ingredient (ng. i.a.) per treated nymph. The Resistance Ratio (RR_50_) was calculated as the quotient of LD_50_ value for field population divided by the LD_50_ value obtained for the SRL.

This study was approved by the Animal Ethics Committee of *Fundação Oswaldo Cruz* (n°. 29/14-1).

## Results

The susceptibility reference lineage presented an LD_50_ of 0.064 ng a.i./nymph treated. The susceptibility profile characterization of *T. sordida* populations revealed RR_50_ values ranging from 2.6 to 6.8. All population presented slope equal or higher than the slope of the SRL, revealing equal or lower heterogeneity in the toxicological response including individuals that resist higher doses of insecticides (Table 1)*.*

**Table 1:** **Toxicological profile to deltamethrin in peridomestic** ***Triatoma sordida*** **from North of Minas Gerais, Brazil**

**Population: municipality**	**Location**	**LD**_**50**_ **(95% CI)**	**RR**_**50**_	***Slope*** **± SD**
		**(ng i.a. / nymph)**	**(95% CI)**	
Uberaba - SRL	-	0.066 (0.055 - 0.077)	1.00 (5.74 - 8.74)	5.315 ± 0.664
Monjolos	Cipó	0.165 (0.114 - 0.234)	2.50 (1.97 - 3.17)	4.878 ± 0.664
Coração de Jesus	Jataí	0.180 (0.131 - 0.234)	2.73 (2.14 - 3.50)	4.804 ± 0.727
Buenópolis	Cercado	0.223 (0.134 - 0.314)	3.39 (2.71 – 4.24)	5.954 ± 1.011
Monte Azul	Tábuas	0.228 (0.207 - 0.249)	3.47 (2.89 - 4.17)	9.975 ± 1.490
Coração de Jesus	Jatobá de Cima	0.235 (0.185 - 0.286)	3.58 (2.74 – 4.66)	4.578 ± 0.656
Monte Azul	Brejinho	0.236 (0.129 - 0.333)	3.59 (2.69 - 4.80)	4.763 ± 0.755
Coração de Jesus	Jatobá	0.269 (0.196 - 0.335)	4.08 (3.94 - 4.74)	4.834 ± 1.002
Monjolos	Tamboril	0.285 (0.248 - 0.329)	4.34 (3.51 - 5.36)	5.864 ± 0.822
Bocaiúva	Félix	0.288 (0.240 - 0.335)	4.38 (3.49 - 5.50)	5.784 ± 0.873
Coração de Jesus	Domingada	0.331 (0.252 - 0.436)	5.03 (4.08 - 6.21)	6.388 ± 0.945
Presidente Juscelino	Mandioca	0.354 (0.263 - 0.453)	5.39 (4.28 – 6.78)	5.629 ± 0.790
Bocaiúva	Chaves	0.372 (0.326 - 0.434)	5.66 (4.57 – 7.00)	7.552 ± 0.999
Bocaiúva	Félix I	0.399 (0.289 - 0.515)	6.06 (4.82 – 7.63)	5.791 ± 0.918
Coração de Jesus	Barriguda	0.465 (0.404 - 0.535)	7.08 (5.74 - 8.74)	6.834 ± 1.033

**SRL:** susceptibility reference lineage; **LD**_**50:**_ 50% lethal dose_;_ **95% CI:** 95% confidence interval; **ng i.a**: nanograms of active ingredient; **RR**_**50:**_ 50% resistance ratio; SD: standard deviation.

## Discussion

Despite *Triatoma sordida* being the most captured species in Central Brazil and that this species is associated with ever increasing frequent reports of persistent reinfestations, making vector control more difficult, there are still few studies that investigate the resistance of these vectors to insecticides.

In this work, the characterization of deltamethrin susceptibility profile in 14 populations of *T. sordida* collected in areas of persistent reinfestation in Northern Minas Gerais, revealed the biggest resistance ratios already identified for populations of *T. sordida* (RR_50_ 2.5 to 7.2). The current knowledge of pyrethroids toxicological profile in Brazilian triatomines is limited. Studies have been done with *T. infestans* [24,25], *Triatoma brasiliensis* [16], *Panstrongylus megistus* [26] and *T. sordida* [21,27,28] showing reduced resistance ratios values (RR_50_ 0.7 to 7.0) when compared with triatomines from Bolivia (RR_50_ 0.62 to 818.0) [25,29-37] and Argentina (RR_50_ 0.9 to 1419.6) [24,29-31,35,36,38-43]. According to OPAS [19], for populations with RR < 5, the alteration of susceptibility observed is related to individual variations, and the maintenance of control activities with the utilized insecticide and continuity with the monitoring activities is recommended. However, the populations with RR_50_ > 5 are considered resistant - Coração de Jesus/Domingada (RR_50_ 5.0), Presidente Juscelino/Mandioca (RR_50_ 5.3), Bocaiúva/Chaves (RR_50_ 5.6), Bocaiúva/Felix I (RR_50_ 6.0) and Coração de Jesus/Barriguda (RR_50_ 7.2). In this case is recommended: 1) to investigate the operational failures in the strategies control vector realized by Chagas Disease Control Program - CDCP; 2) to change the insecticide used for CDCP by other, with distinct mode of action, 3) to continue the susceptibility monitoring studies on the timeline.

In fact, we know very little about the real impact of the resistance ratios obtained in the laboratory bioassays on the effectiveness of the vector control activities in the field. Thus, we prefer to refer to the populations with RR > 5 as populations with altered susceptibility. For these populations, the realization of laboratory and field trials, simultaneous and complementary, permitting the evaluation of both, is recommended. These trials will permit establishment beyond conceptual cut-off points for phenotype resistance, define operational cut-off points that permit the adoption of intervention measures in opportune moments by reverting the resistance frame, not detrimental to the few available insecticides for triatomine density control. It is highlighted here that the continued pressure with insecticide demands that evaluations of this type be made on a continuous timeline, since the population profiles can be altered.

The different deltamethrin susceptibility profile observed in populations of distinct locations, nevertheless belonging to the same municipalities (ex. Coração de Jesus/Jataí RR_50_ 2.7 and Coração de Jesus/Barriguda RR_50_ 7.2), reinforce the complexity of the resistance phenotype, not only at the macrogeographical level, but at the microgeographical level. Different toxicity of deltamethrin was detected between dwellings of Chaco province, accounting for both susceptible and resistant houses within the same locality in *T. infetans* populations [44]. Deltatmethrin appears not to present homogenous effects over different populations, suggesting independent selection processes facing pressure with the insecticide.

Slope values have been used as indicators of population heterogeneity. High slope values are related to low genetic variation, whereas populations in process of selection and thus showing genetic variation relate to less step slopes (when compared to SRL slope) [45]. Of the 14 studied populations, all populations presented equal or higher slope to that of the LRS, suggesting the lack or reduced possibility of deltamethrin toxicological profile change on the timeline should be considered, when submitted to the continuous pressure with insecticide.

With the decentralization of the Chagas Disease Control Program in 1999 [46] a growth in demobilization of the vector control activities for CD were observed. The inexistence of career plans and the low salaries of health agents have reflected in a large rotation of these professionals, which without supervision on the field have developed their activities with questionable quality. In addition to this, with the Certification of the Interruption of Chagas disease by *Triatoma infestans* in Brazil in 2006 [3] a reduction in the priority of the control actions of CD was confirmed, connected to the loss of recognizing the importance of the disease faced with the emergencies of other endemics. This situation may directly reflect on the sustainability of the control levels reached, fruits of decades of effort by agents and health managers.

In this context, the alteration of the susceptibility observed in this work’s study populations may be related to operational failures, caused by: 1) the lack of insecticide efficiency related to the poor quality of active ingredients and/or inadequate formulation; 2) operational failures related to the lack of training of endemic agents [17], 3) environmental conditions, mainly in peridomicile, responsible for the more accelerated degradation of the insecticide [47-49]; 4) complexity of peridomiciles of the region tied to the behavioral characteristics of the *T. sordida* [14,17,50]; and 5) spraying cycle discontinuity, for administrative and budget related reasons [49,51-53]. All of these factors pointed out, isolated or associated, expose the triatomine to sub-lethal doses selecting the least susceptible.

Beyond this, the study area of this work presents overlapping with the dengue and leishmaniasis endemics. Both programs execute simultaneously and without coordination among themselves, their vector chemical control activities, each one meeting their specific characteristics. Together with this fact, the utilization of agricultural and domestic insecticides signifies an exacerbated chemical pressure over the triatomine populations of the region, which can contribute to the indiscriminate and unwanted increase of insecticide resistance [17].

## Conclusions

Considering the different toxicological profiles observed in the populations of *T.sordida* studied in this work, associated with the large ecological valence of this triatomine species, the necessity of more in-depth studies, investigating the real impact of the RR_50_ on the vector control activities in the field, is reinforced. The evaluation of this information, considering the environmental conditions into which the triatomine populations find themselves inserted and the genetic variability of the same will permit the establishment of rational and feasible strategies for the vector control of this specie.
